# The genome sequence of the Essex Skipper butterfly, *Thymelicus lineola *(Ochsenheimer, 1808)

**DOI:** 10.12688/wellcomeopenres.22847.1

**Published:** 2024-08-12

**Authors:** Konrad Lohse, Roger Vila, Alex Hayward, Dominik R. Laetsch, Simon Harnqvist, Wellcome Sanger Institute Tree of Life Management, Samples and Laboratory team, Wellcome Sanger Institute Scientific Operations: Sequencing Operations, Wellcome Sanger Institute Tree of Life Core Informatics team, Tree of Life Core Informatics collective, Darwin Tree of Life Consortium

**Affiliations:** 1 Institute of Ecology and Evolution, University of Edinburgh, Edinburgh, Scotland, UK; 2 Institut de Biologia Evolutiva, CSIC - Universitat Pompeu Fabra, Barcelona, Spain; 3 Department of Biosciences, University of Exeter, Penryn, England, UK

**Keywords:** Thymelicus lineola, Essex Skipper butterfly, genome sequence, chromosomal, Lepidoptera

## Abstract

We present a genome assembly from an individual female Essex Skipper butterfly, *Thymelicus lineola* (Arthropoda; Insecta; Lepidoptera; Hesperiidae). The genome sequence spans 511.80 megabases. Most of the assembly is scaffolded into 30 chromosomal pseudomolecules, including the Z and W sex chromosomes. The mitochondrial genome has also been assembled and is 17.24 kilobases in length.

## Species taxonomy

Eukaryota; Opisthokonta; Metazoa; Eumetazoa; Bilateria; Protostomia; Ecdysozoa; Panarthropoda; Arthropoda; Mandibulata; Pancrustacea; Hexapoda; Insecta; Dicondylia; Pterygota; Neoptera; Endopterygota; Amphiesmenoptera; Lepidoptera; Glossata; Neolepidoptera; Heteroneura; Ditrysia; Obtectomera; Hesperioidea; Hesperiidae; Hesperiinae; Hesperiini; *Thymelicu*s; *Thymelicus lineola* (Ochsenheimer, 1808) (NCBI:txid218773).

## Background

The Essex Skipper ( *Thymelicus lineola*), also known as the European Skipper, is a widely distributed butterfly found from northern Finland to North Africa, and from Central Asia to Great Britain. It also occurs in Canada and the United States due to an accidental introduction in 1910 ( Saunders, 1916). In Britain, it is found in most of southeast England, the Midlands, and in eastern parts of Wales. It is absent from Scotland and also from Ireland. The Essex Skipper is considered at low risk of extinction; on the IUCN Red list (Europe) it is listed as being of Least Concern. In Britain, its range has more than doubled in recent decades ( Butterfly Conservation, no date).

The species is a habitat generalist with substantial dispersal ability ( Louy *et al.*, 2007). The main food plant in the UK is Cock’s-foot ( *Dactylis glomerata)* but it is also feeds on several other grasses, including Creeping Soft-grass ( *Holcus mollis*), Common Couch ( *Elytrigia repens*), Timothy ( *Phleum pratense*), Meadow Foxtail ( *Alopecurus pratensis*), False Brome ( *Brachypodium sylvaticum*), and Tor-grass ( *Brachypodium pinnatum*) and – rarely – Yorkshire-fog ( *Holcus lanatus*) ( Butterfly Conservation, no date). The species is univoltine with adults flying from May to August, depending on locality.

Previous research indicates that the Essex Skipper contains three highly diverged mitochondrial lineages in the West Palaearctic, each corresponding to a different glacial refugium in a Mediterranean Peninsula (Iberian, Italian and Balkan peninsulas). Western Europe and Great Britain were apparently colonised post-glacially from Italy, and Eastern Europe from the Balkans ( Dapporto *et al.*, 2022). The source for the introduction to the Nearctic corresponds genetically to the Italian/West European/Great Britain lineage ( D’Ercole *et al.*, 2022). While local population structure estimated with allozymes was lower in *T. lineola* than in its two congeners in Europe *T. acteon* and *T. sylvestris* ( Engler *et al.*, 2014; Louy *et al.*, 2007), genomic analyses show that *T. lineola* has substantial genetic diversity, above the average in butterflies ( Mackintosh *et al.*, 2019). The Essex Skipper has 29 chromosomes ( Federley, 1938; Larsen, 1975). The genome sequence will help understand the population structure and history of *T. lineola* and its relationship with its congeners.

## Genome sequence report

**Figure 1.**
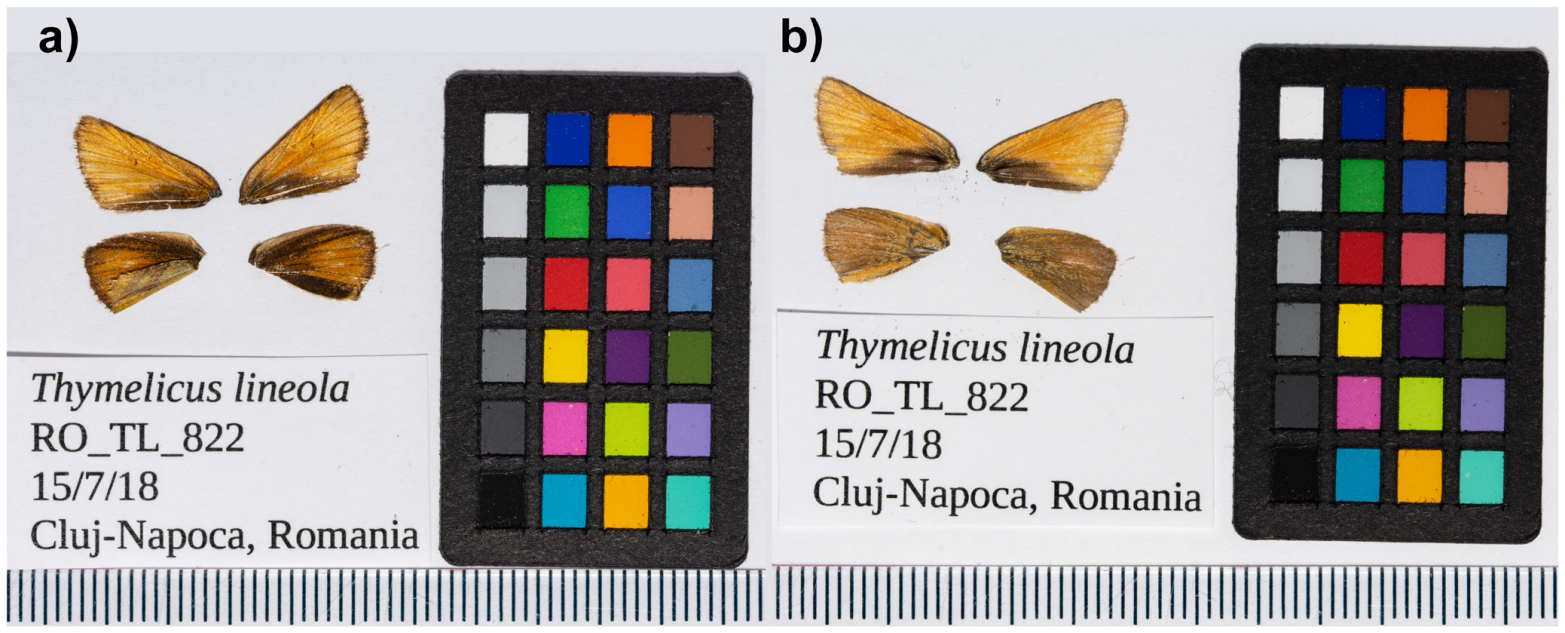
Photographs of forewings and hindwings of the *Thymelicus* lineola (ilThyLine1 specimen RO_TL_822 used for genome sequencing. **a**) Dorsal and **b**) ventral surface views of wings from the specimen.

The genome of an adult female *Thymelicus lineola* ( Figure 1) was sequenced using Pacific Biosciences single-molecule HiFi long reads, generating a total of 9.13 Gb (gigabases) from 0.95 million reads, providing approximately 18-fold coverage. Primary assembly contigs were scaffolded with chromosome conformation Hi-C data, which produced 61.38 Gbp from 406.51 million reads, yielding an approximate coverage of 120-fold. Specimen and sequencing information is summarised in Table 1.

**Table 1.** Specimen and sequencing data for *Thymelicus lineola*.

Project information			
**Study title**	Thymelicus lineola (Essex skipper)		
**Umbrella BioProject**	PRJEB59398		
**Species**	*Thymelicus lineola*		
**BioSample**	SAMEA7523301		
**NCBI taxonomy ID**	218773		
Specimen information			
**Technology**	**ToLID**	**BioSample accession**	**Organism part**
**PacBio long read sequencing**	ilThyLine1	SAMEA7523397	Whole organism
**Hi-C sequencing**	ilThyLine1	SAMEA7523397	Whole organism
Sequencing information			
**Platform**	**Run accession**	**Read count**	**Base count (Gb)**
**Hi-C HiSeq X Ten**	ERR10851543	4.07e+08	61.38
**PacBio Sequel II**	ERR10925358	9.52e+05	9.13

**Figure 2.**
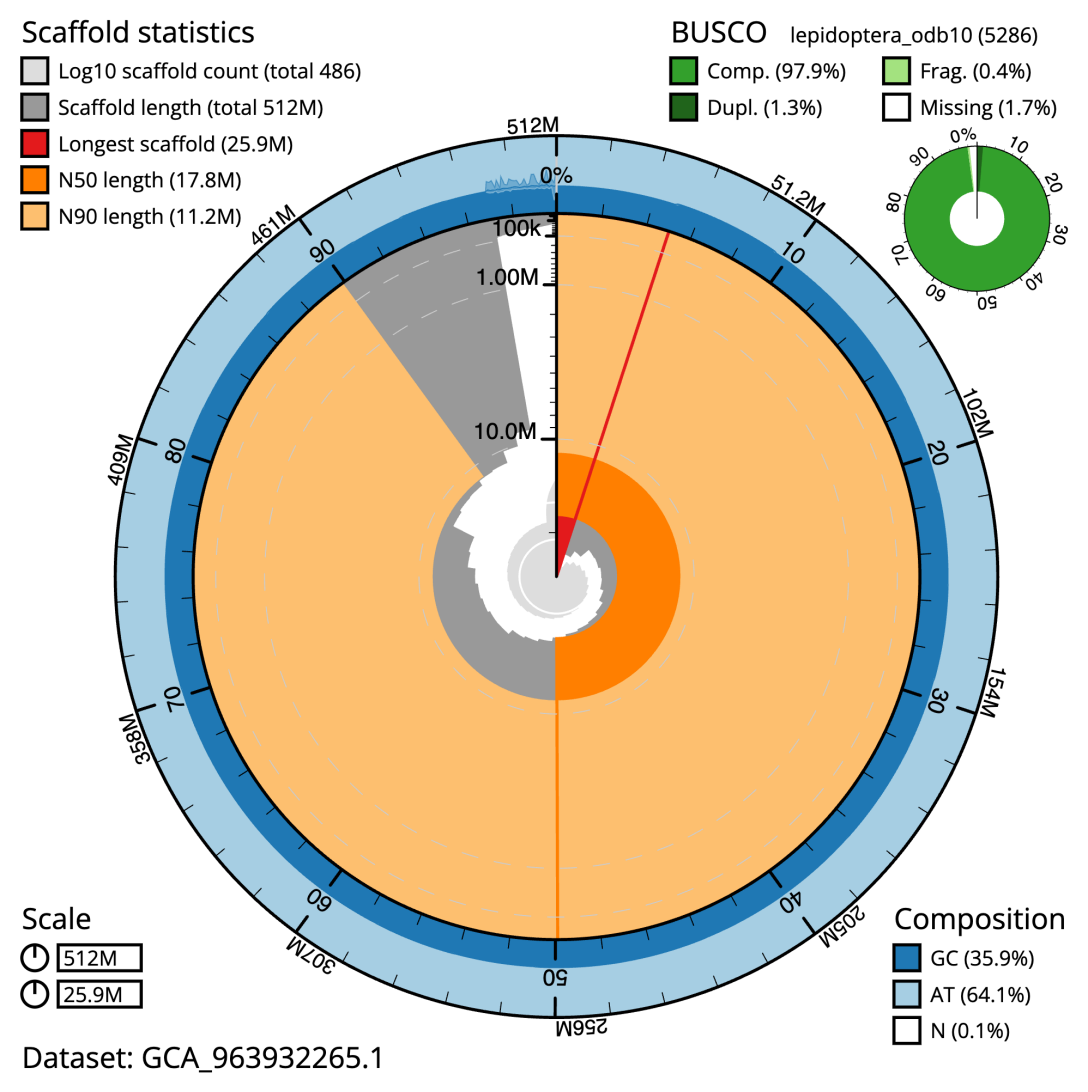
Genome assembly of *Thymelicus lineola*, ilThyLine1.1: metrics. The BlobToolKit snail plot shows N50 metrics and BUSCO gene completeness. The main plot is divided into 1,000 size-ordered bins around the circumference with each bin representing 0.1% of the 511,826,406 bp assembly. The distribution of scaffold lengths is shown in dark grey with the plot radius scaled to the longest scaffold present in the assembly (25,889,543 bp, shown in red). Orange and pale-orange arcs show the N50 and N90 scaffold lengths (17,791,929 and 11,247,964 bp), respectively. The pale grey spiral shows the cumulative scaffold count on a log scale with white scale lines showing successive orders of magnitude. The blue and pale-blue area around the outside of the plot shows the distribution of GC, AT and N percentages in the same bins as the inner plot. A summary of complete, fragmented, duplicated and missing BUSCO genes in the lepidoptera_odb10 set is shown in the top right. An interactive version of this figure is available at https://blobtoolkit.genomehubs.org/view/Thymelicus_lineola/dataset/GCA_963932265.1/snail.

**Figure 3.**
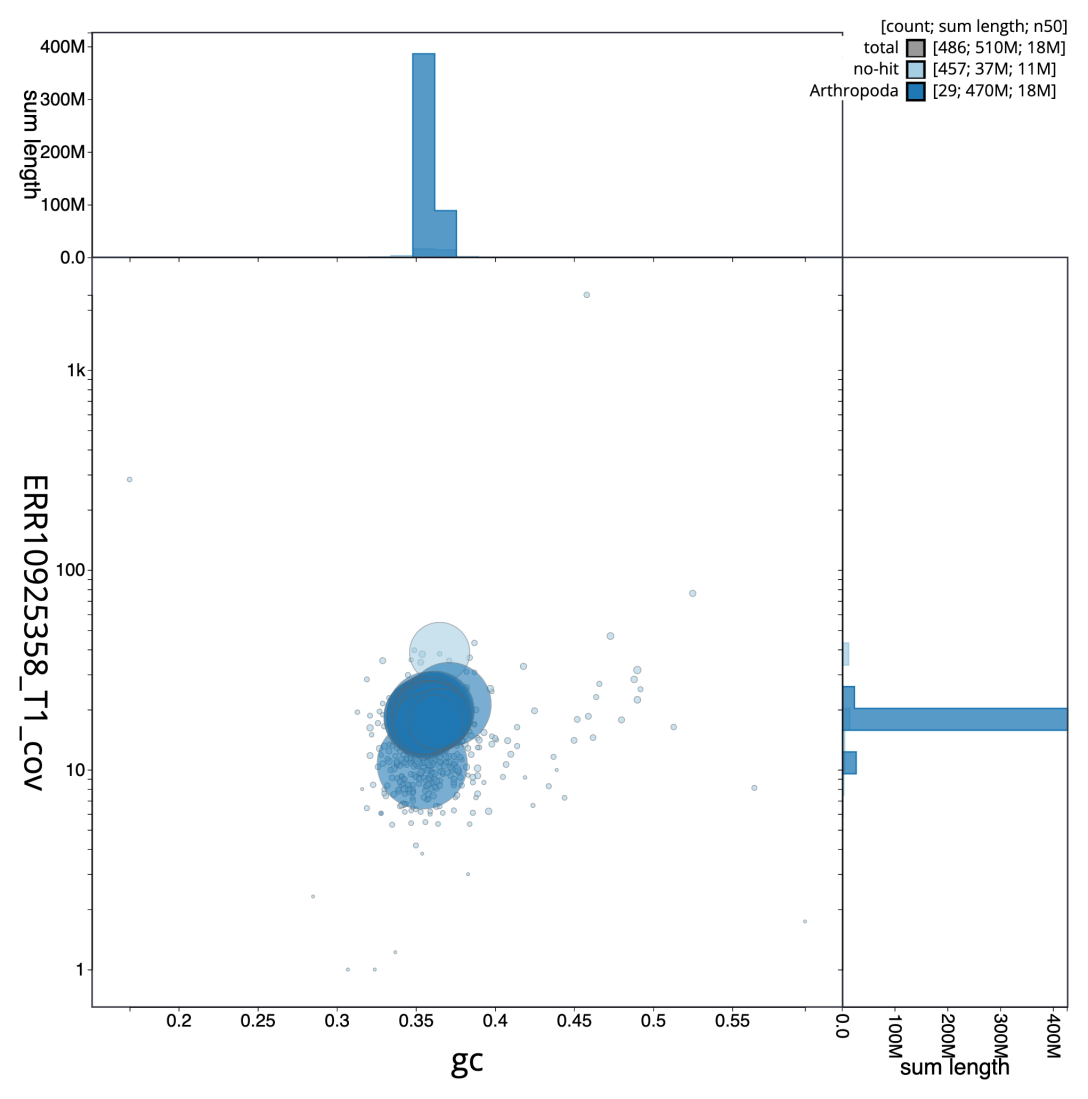
Genome assembly of *Thymelicus lineola*, ilThyLine1.1: BlobToolKit GC-coverage plot. Sequences are coloured by phylum. Circles are sized in proportion to sequence length. Histograms show the distribution of sequence length sum along each axis. An interactive version of this figure is available at https://blobtoolkit.genomehubs.org/view/Thymelicus_lineola/dataset/GCA_963932265.1/blob.

**Figure 4.**
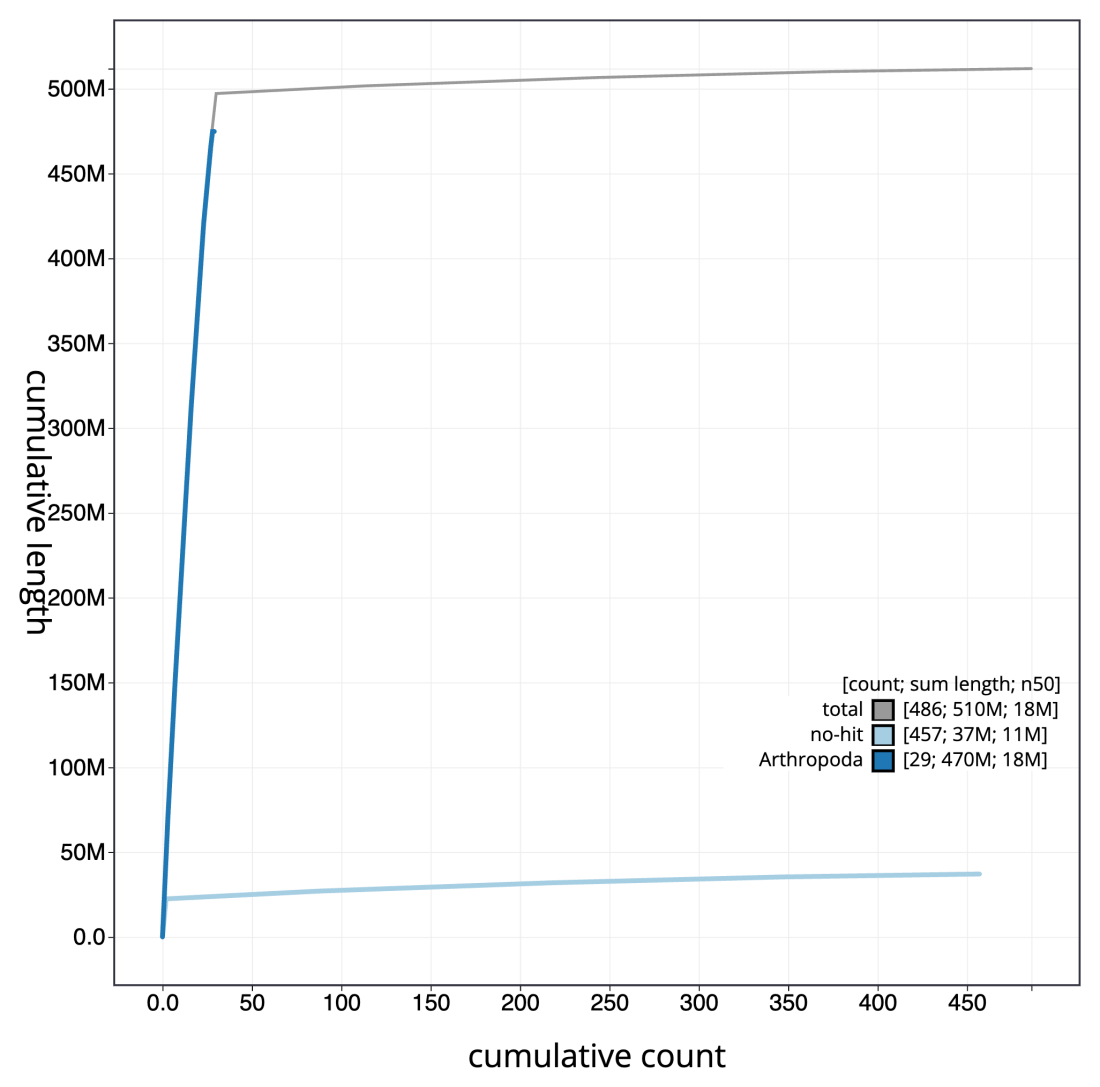
Genome assembly of *Thymelicus lineola* ilThyLine1.1: BlobToolKit cumulative sequence plot. The grey line shows cumulative length for all sequences. Coloured lines show cumulative lengths of sequences assigned to each phylum using the buscogenes taxrule. An interactive version of this figure is available at https://blobtoolkit.genomehubs.org/view/Thymelicus_lineola/dataset/GCA_963932265.1/cumulative.

**Figure 5.**
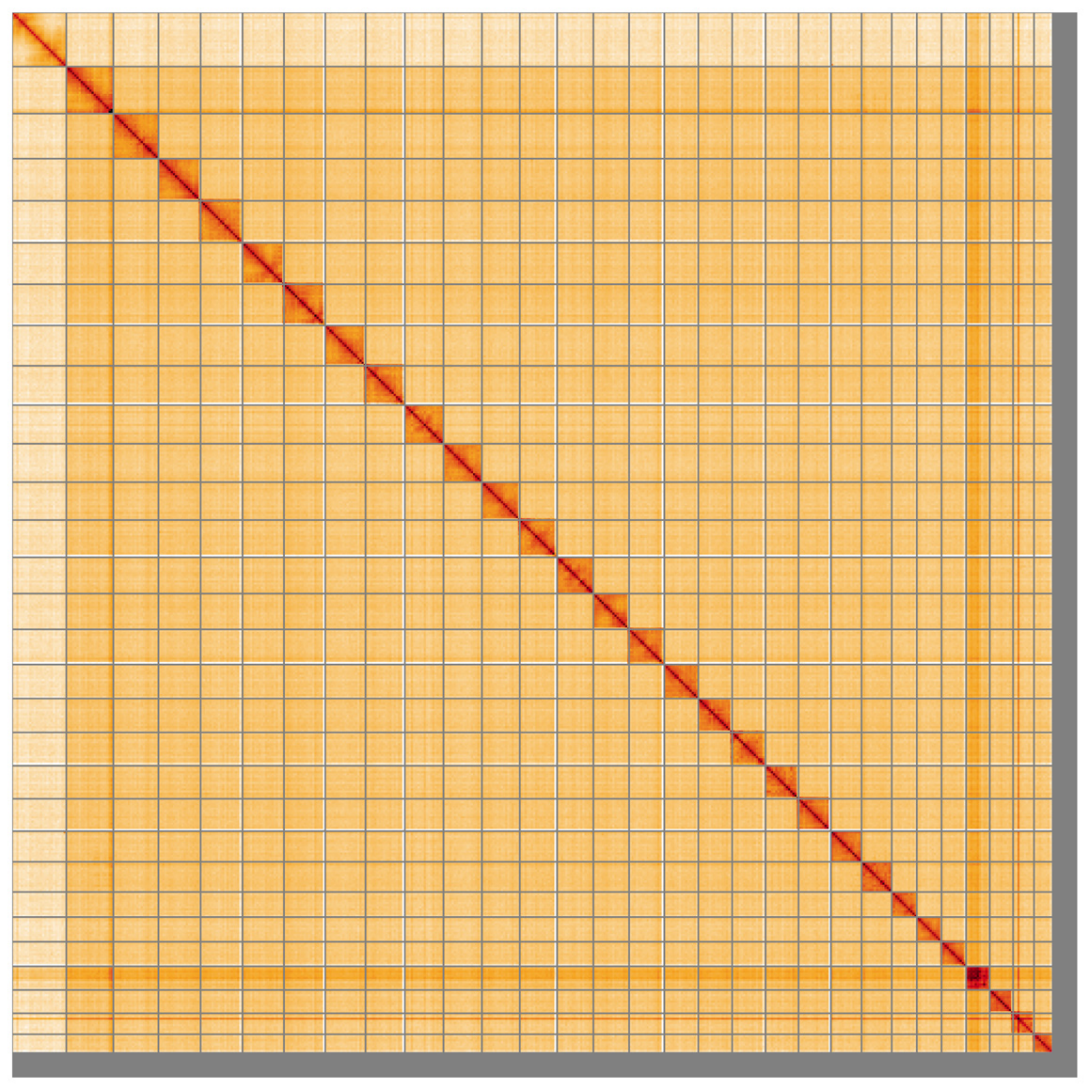
Genome assembly of *Thymelicus lineola* ilThyLine1.1: Hi-C contact map of the ilThyLine1.1 assembly, visualised using HiGlass. Chromosomes are shown in order of size from left to right and top to bottom. An interactive version of this figure may be viewed at https://genome-note-higlass.tol.sanger.ac.uk/l/?d=IVDpKRXFTyupA1roZBe5uQ.

Manual assembly curation corrected 360 missing joins or mis-joins and 362 haplotypic duplications, reducing the assembly length by 9.69% and the scaffold number by 46.65%, and increasing the scaffold N50 by 17.53%. The final assembly has a total length of 511.80 Mb in 485 sequence scaffolds with a scaffold N50 of 17.8 Mb ( Table 2). The total count of gaps in the scaffolds is 1,582. The snail plot in Figure 2 provides a summary of the assembly statistics, while the distribution of assembly scaffolds on GC proportion and coverage is shown in Figure 3. The cumulative assembly plot in Figure 4 shows curves for subsets of scaffolds assigned to different phyla. Most (97.12%) of the assembly sequence was assigned to 30 chromosomal-level scaffolds, representing 28 autosomes and the Z and W sex chromosomes. Chromosome-scale scaffolds confirmed by the Hi-C data are named in order of size ( Figure 5; Table 3). Chromosome Z identified by coverage and alignment to *Thymelicus sylvestris* (GCA_911387775.1) ( Hayward *et al.*, 2022). The order and orientation of scaffolds in Chromosome W is uncertain. While not fully phased, the assembly deposited is of one haplotype. Contigs corresponding to the second haplotype have also been deposited. The mitochondrial genome was also assembled and can be found as a contig within the multifasta file of the genome submission.

**Table 2.** Genome assembly data for *Thymelicus lineola*, ilThyLine1.1.

Genome assembly		
Assembly name	ilThyLine1.1	
Assembly accession	GCA_963932265.1	
*Accession of alternate haplotype*	*GCA_963932395.1*	
Span (Mb)	511.80	
Number of contigs	2,068	
Contig N50 length (Mb)	0.5	
Number of scaffolds	485	
Scaffold N50 length (Mb)	17.8	
Longest scaffold (Mb)	25.89	
Assembly metrics *		*Benchmark*
Consensus quality (QV)	60.5	*≥ 50*
*k*-mer completeness	100.0%	*≥ 95%*
BUSCO **	C:97.8%[S:96.5%,D:1.3%], F:0.5%,M:1.7%,n:5,286	*C ≥ 95%*
Percentage of assembly mapped to chromosomes	97.12%	*≥ 95%*
Sex chromosomes	ZW	*localised homologous pairs*
Organelles	Mitochondrial genome: 17.24 kb	*complete single alleles*

* Assembly metric benchmarks are adapted from column VGP-2020 of “Table 1: Proposed standards and metrics for defining genome assembly quality” from Rhie *et al.* (2021). ** BUSCO scores based on the lepidoptera_odb10 BUSCO set using version 5.4.3. C = complete [S = single copy, D = duplicated], F = fragmented, M = missing, n = number of orthologues in comparison. A full set of BUSCO scores is available at https://blobtoolkit.genomehubs.org/view/Thymelicus_lineola/dataset/GCA_963932265.1/busco.

**Table 3.** Chromosomal pseudomolecules in the genome assembly of *Thymelicus lineola*, ilThyLine1.

INSDC accession	Name	Length (Mb)	GC%
OZ010681.1	1	22.48	37.0
OZ010682.1	2	21.52	36.0
OZ010683.1	3	20.14	36.0
OZ010684.1	4	20.05	35.5
OZ010685.1	5	19.78	36.0
OZ010686.1	6	19.7	36.0
OZ010687.1	7	19.28	35.5
OZ010688.1	8	18.77	35.5
OZ010689.1	9	18.46	35.5
OZ010690.1	10	18.33	35.5
OZ010691.1	11	18.12	36.0
OZ010692.1	12	17.79	36.0
OZ010693.1	13	17.37	35.5
OZ010694.1	14	17.07	36.0
OZ010695.1	15	16.84	35.5
OZ010696.1	16	16.22	36.0
OZ010697.1	17	16.19	36.0
OZ010698.1	18	15.94	36.0
OZ010699.1	19	15.75	36.0
OZ010700.1	20	15.47	36.0
OZ010701.1	21	14.62	36.0
OZ010702.1	22	14.41	36.0
OZ010703.1	23	12.01	35.5
OZ010704.1	24	11.8	35.5
OZ010705.1	25	11.74	36.5
OZ010707.1	26	11.15	36.0
OZ010708.1	27	10.05	36.5
OZ010709.1	28	8.91	36.5
OZ010706.1	W	11.25	36.5
OZ010680.1	Z	25.89	35.5
OZ010710.1	MT	0.02	17.0

The estimated Quality Value (QV) of the final assembly is 60.5 with *k*-mer completeness of 100.0%, and the assembly has a BUSCO v5.4.3 completeness of 97.8% (single = 96.5%, duplicated = 1.3%), using the lepidoptera_odb10 reference set ( *n* = 5,286).

Metadata for specimens, BOLD barcode results, spectra estimates, sequencing runs, contaminants and pre-curation assembly statistics are given at https://links.tol.sanger.ac.uk/species/218773.

## Methods

### Sample acquisition and nucleic acid extraction

A female adult *Thymelicus lineola* (specimen ID SAN0000863, ToLID ilThyLine1) was collected from Romania (latitude 46.8, longitude 23.5) on 2018-07-15 by daytime using a handnet. The collectors were Konrad Lohse, Alex Hayward, Dominik Laetsch (all from the University of Edinburgh) and Roger Vila (University of Exeter). The specimen was formally identified by Roger Vila. It was snap-frozen from live in a dry shipper.

The workflow for high molecular weight (HMW) DNA extraction at the Wellcome Sanger Institute (WSI) Tree of Life Core Laboratory includes a sequence of core procedures: sample preparation; sample homogenisation, DNA extraction, fragmentation, and clean-up. In sample preparation, the ilThyLine1 sample was weighed and dissected on dry ice ( Jay *et al.*, 2023). Tissue from the whole organism was homogenised using a PowerMasher II tissue disruptor ( Denton *et al.*, 2023a).

HMW DNA was extracted at the WSI Scientific Operations core using the Automated MagAttract v2 protocol ( Oatley *et al.*, 2023). The DNA was sheared into an average fragment size of 12–20 kb in a Megaruptor 3 system with speed setting 31 ( Bates *et al.*, 2023). Sheared DNA was purified by solid-phase reversible immobilisation ( Strickland *et al.*, 2023): in brief, the method employs AMPure PB beads to eliminate shorter fragments and concentrate the DNA. The concentration of the sheared and purified DNA was assessed using a Nanodrop spectrophotometer and Qubit Fluorometer using the Qubit dsDNA High Sensitivity Assay kit. Fragment size distribution was evaluated by running the sample on the FemtoPulse system.

Protocols developed by the WSI Tree of Life laboratory are publicly available on protocols.io ( Denton *et al.*, 2023b).

### Sequencing

Pacific Biosciences HiFi circular consensus DNA sequencing libraries were constructed according to the manufacturers’ instructions. DNA sequencing was performed by the Scientific Operations core at the WSI on a Pacific Biosciences Sequel II instrument. Hi-C data were also generated from whole organism tissue of ilThyLine1 using the Arima-HiC v2 kit. The Hi-C sequencing was performed using paired-end sequencing with a read length of 150 bp on the HiSeq X Ten instrument.

### Genome assembly, curation and evaluation

***Assembly***

The original assembly of HiFi reads was performed using Hifiasm ( Cheng *et al.*, 2021) with the --primary option. Haplotypic duplications were identified and removed with purge_dups ( Guan *et al.*, 2020). Hi-C reads were further mapped with bwa-mem2 ( Vasimuddin *et al.*, 2019) to the primary contigs, which were further scaffolded using the provided Hi-C data ( Rao *et al.*, 2014) in YaHS ( Zhou *et al.*, 2023) using the --break option. Scaffolded assemblies were evaluated using Gfastats ( Formenti *et al.*, 2022), BUSCO ( Manni *et al.*, 2021) and MERQURY.FK ( Rhie *et al.*, 2020).

The mitochondrial genome was assembled using MitoHiFi ( Uliano-Silva *et al.*, 2023), which runs MitoFinder ( Allio *et al.*, 2020) and uses these annotations to select the final mitochondrial contig and to ensure the general quality of the sequence.

***Assembly curation***

The assembly was decontaminated using the Assembly Screen for Cobionts and Contaminants (ASCC) pipeline (article in preparation). Flat files and maps used in curation were generated in TreeVal ( Pointon *et al.*, 2023). Manual curation was primarily conducted using PretextView ( Harry, 2022), with additional insights provided by JBrowse2 ( Diesh *et al.*, 2023) and HiGlass ( Kerpedjiev *et al.*, 2018). Scaffolds were visually inspected and corrected as described by Howe *et al*. (2021). Any identified contamination, missed joins, and mis-joins were corrected, and duplicate sequences were tagged and removed. The sex chromosome was identified by means of synteny. An AGP file was generated from the curated Pretext map to describe the order, orientation, and gaps between contigs. The TPF file was then created using the AGP file and the original fasta and TPF files. Chromosomes on the resolved map were painted, and the curated data was processed to produce the final assembly using the multi_join.py script. The entire process is documented at https://gitlab.com/wtsi-grit/rapid-curation (article in preparation).

***Evaluation of the final assembly***

The final assembly was post-processed and evaluated with the three Nextflow ( Di Tommaso *et al.*, 2017) DSL2 pipelines “sanger-tol/readmapping” ( Surana *et al.*, 2023a), “sanger-tol/genomenote” ( Surana *et al.*, 2023b), and “sanger-tol/blobtoolkit” ( Muffato *et al.*, 2024). The pipeline sanger-tol/readmapping aligns the Hi-C reads with bwa-mem2 ( Vasimuddin *et al.*, 2019) and combines the alignment files with SAMtools ( Danecek *et al.*, 2021). The sanger-tol/genomenote pipeline transforms the Hi-C alignments into a contact map with BEDTools ( Quinlan & Hall, 2010) and the Cooler tool suite ( Abdennur & Mirny, 2020), which is then visualised with HiGlass ( Kerpedjiev *et al.*, 2018). It also provides statistics about the assembly with the NCBI datasets ( Sayers *et al.*, 2024) report, computes *k*-mer completeness and QV consensus quality values with FastK and MERQURY.FK, and a completeness assessment with BUSCO ( Manni *et al.*, 2021).

The sanger-tol/blobtoolkit pipeline is a Nextflow port of the previous Snakemake Blobtoolkit pipeline ( Challis *et al.*, 2020). It aligns the PacBio reads with SAMtools and minimap2 ( Li, 2018) and generates coverage tracks for regions of fixed size. In parallel, it queries the GoaT database ( Challis *et al.*, 2023) to identify all matching BUSCO lineages to run BUSCO ( Manni *et al.*, 2021). For the three domain-level BUSCO lineages, the pipeline aligns the BUSCO genes to the Uniprot Reference Proteomes database ( Bateman *et al.*, 2023) with DIAMOND ( Buchfink *et al.*, 2021) blastp. The genome is also split into chunks according to the density of the BUSCO genes from the closest taxonomically lineage, and each chunk is aligned to the Uniprot Reference Proteomes database with DIAMOND blastx. Genome sequences that have no hit are then chunked with seqtk and aligned to the NT database with blastn ( Altschul *et al.*, 1990). All those outputs are combined with the blobtools suite into a blobdir for visualisation.

The genome assembly and evaluation pipelines were developed using the nf-core tooling ( Ewels *et al.*, 2020), use MultiQC ( Ewels *et al.*, 2016), and make extensive use of the Conda package manager, the Bioconda initiative ( Grüning *et al.*, 2018), the Biocontainers infrastructure ( da Veiga Leprevost *et al.*, 2017), and the Docker ( Merkel, 2014) and Singularity ( Kurtzer *et al.*, 2017) containerisation solutions.

Table 4 contains a list of relevant software tool versions and sources.

**Table 4.** Software tools: versions and sources.

Software tool	Version	Source
BEDTools	2.30.0	https://github.com/arq5x/bedtools2
BLAST	2.14.0	ftp://ftp.ncbi.nlm.nih.gov/blast/executables/blast+/
BlobToolKit	4.3.7	https://github.com/blobtoolkit/blobtoolkit
BUSCO	5.4.3 and 5.5.0	https://gitlab.com/ezlab/busco
bwa-mem2	2.2.1	https://github.com/bwa-mem2/bwa-mem2
Cooler	0.8.11	https://github.com/open2c/cooler
DIAMOND	2.1.8	https://github.com/bbuchfink/diamond
fasta_windows	0.2.4	https://github.com/tolkit/fasta_windows
FastK	427104ea91c78c3b8b8b49f1a7d6bbeaa869ba1c	https://github.com/thegenemyers/FASTK
Gfastats	1.3.6	https://github.com/vgl-hub/gfastats
GoaT CLI	0.2.3	https://github.com/genomehubs/goat-cli
Hifiasm	0.16.1-r375	https://github.com/chhylp123/hifiasm
HiGlass	44086069ee7d4d3f6f3f0012569789ec138f42b84 aa44357826c0b6753eb28de	https://github.com/higlass/higlass
Merqury.FK	d00d98157618f4e8d1a9190026b19b471055b22e	https://github.com/thegenemyers/MERQURY.FK
MitoHiFi	2	https://github.com/marcelauliano/MitoHiFi
MultiQC	1.14, 1.17, and 1.18	https://github.com/MultiQC/MultiQC
NCBI Datasets	15.12.0	https://github.com/ncbi/datasets
Nextflow	23.04.0-5857	https://github.com/nextflow-io/nextflow
PretextView	0.2	https://github.com/sanger-tol/PretextView
purge_dups	1.2.5	https://github.com/dfguan/purge_dups
samtools	1.16.1, 1.17, and 1.18	https://github.com/samtools/samtools
sanger-tol/ascc	-	https://github.com/sanger-tol/ascc
sanger-tol/ genomenote	1.1.1	https://github.com/sanger-tol/genomenote
sanger-tol/ readmapping	1.2.1	https://github.com/sanger-tol/readmapping
Seqtk	1.3	https://github.com/lh3/seqtk
Singularity	3.9.0	https://github.com/sylabs/singularity
TreeVal	1.0.0	https://github.com/sanger-tol/treeval
YaHS	1.2a.2	https://github.com/c-zhou/yahs

### Wellcome Sanger Institute – Legal and Governance

The materials that have contributed to this genome note have been supplied by a Darwin Tree of Life Partner. The submission of materials by a Darwin Tree of Life Partner is subject to the **‘Darwin Tree of Life Project Sampling Code of Practice’**, which can be found in full on the Darwin Tree of Life website here. By agreeing with and signing up to the Sampling Code of Practice, the Darwin Tree of Life Partner agrees they will meet the legal and ethical requirements and standards set out within this document in respect of all samples acquired for, and supplied to, the Darwin Tree of Life Project.

Further, the Wellcome Sanger Institute employs a process whereby due diligence is carried out proportionate to the nature of the materials themselves, and the circumstances under which they have been/are to be collected and provided for use. The purpose of this is to address and mitigate any potential legal and/or ethical implications of receipt and use of the materials as part of the research project, and to ensure that in doing so we align with best practice wherever possible. The overarching areas of consideration are:

●   Ethical review of provenance and sourcing of the material

●   Legality of collection, transfer and use (national and international) 

Each transfer of samples is further undertaken according to a Research Collaboration Agreement or Material Transfer Agreement entered into by the Darwin Tree of Life Partner, Genome Research Limited (operating as the Wellcome Sanger Institute), and in some circumstances other Darwin Tree of Life collaborators.

## Data Availability

European Nucleotide Archive: *Thymelicus lineola* (Essex skipper). Accession number PRJEB59398; https://identifiers.org/ena.embl/PRJEB59398 ( Wellcome Sanger Institute, 2024). The genome sequence is released openly for reuse. The *Thymelicus lineola* genome sequencing initiative is part of the Darwin Tree of Life (DToL) project. All raw sequence data and the assembly have been deposited in INSDC databases. The genome will be annotated using available RNA-Seq data and presented through the Ensembl pipeline at the European Bioinformatics Institute. Raw data and assembly accession identifiers are reported in Table 1 and Table 2.

## Author information

Members of the Wellcome Sanger Institute Tree of Life Management, Samples and Laboratory team are listed here: https://doi.org/10.5281/zenodo.12162482.

Members of Wellcome Sanger Institute Scientific Operations: Sequencing Operations are listed here: https://doi.org/10.5281/zenodo.12165051.

Members of the Wellcome Sanger Institute Tree of Life Core Informatics team are listed here: https://doi.org/10.5281/zenodo.12160324.

Members of the Tree of Life Core Informatics collective are listed here: https://doi.org/10.5281/zenodo.12205391.

Members of the Darwin Tree of Life Consortium are listed here: https://doi.org/10.5281/zenodo.4783558.
