# Supplementary material for: The genome sequence of the Essex Skipper butterfly, Thymelicus lineola (Ochsenheimer, 1808)
Source: Wellcome Open Res. 2024 Aug 12;9:452. [Version 1] doi: 10.12688/wellcomeopenres.22847.1 (PMC11480711; doi:10.12688/wellcomeopenres.22847.1)
Supplement: Peer review history

# Reviewer report: Reviewer response for version 1

**DOI:** 10.21956/wellcomeopenres.25155.r98788

**Published:** 2024-10-15

**Authors:** Shuichiro Tomita, Tsuneyuki Tatsuke

**Affiliations:** 1 Silkworm Research Group Institution: Institute of Agrobiological Sciences, NARO, Tsukuba, Japan, Tsukuba, Ibaraki, Japan

This is a well written paper.  The methods employed are appropriate, the results are clean and clear. The quality of the genome assembly presented well complies with today’s standard and the Hi-C results guarantee its quality. I have no comments or instructions. I suggest proceeding with the indexing of this article.

Are sufficient details of methods and materials provided to allow replication by others?

Yes

Is the rationale for creating the dataset(s) clearly described?

Yes

Are the datasets clearly presented in a useable and accessible format?

Yes

Are the protocols appropriate and is the work technically sound?

Yes

Reviewer Expertise:

developmental and evlotulonal biology of insects

We confirm that we have read this submission and believe that we have an appropriate level of expertise to confirm that it is of an acceptable scientific standard.

# Reviewer report: Reviewer response for version 1

**DOI:** 10.21956/wellcomeopenres.25155.r99562

**Published:** 2024-10-15

**Authors:** Alena Sucháčková Bartonova

**Affiliations:** 1 Institute of Entomology, Biology Centre of the Czech Academy of Sciences, České Budějovice, Czech Republic

This is a note for the genome of *Thymelicus lineola* from Darwin Tree of Life.

**Minor comments:**

 Background - The specimen was collected in Romania and the host plants and distribution are listed for the UK. Please check other sources (e.g., Collins butterfly guide) and generalize these statements to Europe or the entire distribution range.

 Sample acquisition - there are errors in the affiliations of the collectors.

Are sufficient details of methods and materials provided to allow replication by others?

Yes

Is the rationale for creating the dataset(s) clearly described?

Yes

Are the datasets clearly presented in a useable and accessible format?

Yes

Are the protocols appropriate and is the work technically sound?

Yes

Reviewer Expertise:

butterflies; biogeography; ecology; conservation; population genetics

I confirm that I have read this submission and believe that I have an appropriate level of expertise to confirm that it is of an acceptable scientific standard.
